# Teaching learners with autism in the South African inclusive classroom: Pedagogic strategies and possibilities

**DOI:** 10.4102/ajod.v11i0.979

**Published:** 2022-06-30

**Authors:** Moleli Nthibeli, Dominic Griffiths, Tanya Bekker

**Affiliations:** 1 Faculty of Humanities, Wits School of Education, University of the Witwatersrand, Johannesburg, South Africa

**Keywords:** inclusive education, inclusive education policy, autism spectrum disorder, inclusive pedagogies, South African inclusive education

## Abstract

**Background:**

Although inclusive education is widely discussed, its implementation has not, arguably, been far-reaching. There remains a lack of specific, targeted approaches towards fully including learners with physical and mental impairments in the educational space.

**Objectives:**

This study investigated the extent of the inclusion of learners with autism spectrum disorder (ASD) in three schools in Johannesburg.

**Method:**

A qualitative interpretivist design was adopted. Teachers who work with learners with ASD were interviewed using open-ended questions. The sampled data were analysed using thematic analysis, making use of both a priori codes and emergent codes that arose from the open-ended questions.

**Results:**

The findings reveal numerous pedagogic strategies such as differentiation, scaffolding, use of visual cues, group work and collaboration that can include learners with ASD in the classroom space.

**Conclusion:**

Teacher collaboration and teacher training are vital in ensuring that learners with ASD are fully included in the classroom space.

## Introduction

Since adopting the Education White Paper 6 on Special Needs Education (EWP6) (Department of Education 2001), inclusive education in South Africa has not made significant developments or seen significant implementation across the country (Department of Education 2016; Kalinnikova Magnusson & Walton 2021; Meiring et al. 2016). Even though South African laws and policies favour inclusive education, the reality is that public, mainstream schools rarely show the will or have the capacity to provide equal educational opportunities to all learner citizens. Engelbrecht et al. (2016:523) state that mainstream schools particularly lack physical facilities, and that there is limited ‘availability of appropriately educated teachers and effective and adequate teaching and learning resources’. This lowers the chances of fostering inclusive education in South Africa in mainstream schools. Mainstream schools are the first level of three types of schools described in EWP6 (Department of Education 2001) for education provisioning in South Africa. They are intended to cater for the diversity of learners in their classrooms, including those with low to moderate support needs. However, without proper infrastructure and sufficient teaching skills, providing this support is challenging.

The second type of school, full-service schools, are schools that are equipped with additional human and material resources to cater for the needs of learners with impairments and those without impairments who are considered to have moderate to high support needs (Department of Education 2010a). Although government intends to convert more mainstream schools into full-service schools, it is not only the responsibility of full-service schools to implement inclusive education. Special education schools are the third level of school placement in the education system for learners identified as having high-level support needs that cannot be accommodated in mainstream or full-service schools. However, despite this tiered schooling, there has been minimal progress in the implementation of inclusive education throughout the education system since the beginning of democracy in South Africa in 1994. Prompt action is necessary to introduce the changes pledged in legislation and policy (Engelbrecht, Smit & Deventer, 2016). Significantly, 2021 was the year in which, across the policy’s 20-year plan, major strides towards inclusive education were supposed to have been made (Department of Education 2001). However, it is evident and undeniable that the quest for inclusive education has fallen far short of its mark, as evidenced by the most recent official report on this objective, which highlights ‘persistent challenges that retard the progress that is being made in the development of an inclusive education and training system’ (Department of Education 2016:6).

The lack of implementation of inclusive education in South Africa is the result of a variety of factors. Studies (Du Plessis 2013; Engelbrecht et al. 2016) have shown that one of the reasons for the slow progress is that inclusive education policy differs markedly from the realities in schools. Donohue and Bornman (2015) point to the EWP6 as an idealistic framework that does not relate meaningfully to the South African context. Moreover, the lack of teacher training is another hindrance to the advancement of inclusion. The transformation of an education system is largely dependent on teachers’ skills, as they are in a position of action where they can practically implement change. The Salamanca Statement (UNESCO 1994) affirms that a prerequisite of inclusive teaching is suitable training in catering for the needs of learners with disabilities. Jansen (2001) explains that teachers prior to 1994 were controlled by a state-enforced, rigid curriculum and were unable to be creative and thorough in their approaches. Regrettably, ‘pedagogies forged under the apartheid regime have not been fundamentally transformed’ (Griffiths & Prozesky 2020:5). South Africa has therefore developed a policy without matching it with the practical aspect of teacher training, and this arguably renders the goal for inclusion unattainable, yet the EWP6 itself states that teachers are the prime stakeholders for the implementation of inclusive education.

The national policy on inclusive education set an objective to reform teacher training and align it according to inclusive educational principles (Department of Education 2001), fulfilling the international recommendation for ‘teacher education programmes’ that ‘address the provision of special needs education in inclusive schools’ (UNESCO 1994:4). Donohue and Bornman (2014) rightly argue that inclusive education is not possible without appropriately trained and resourced teachers. This is crucial, as the neglect of teachers and the undermining of their role in enabling transformative education has, according to Makhalemele and Payne-van Staden (2018), arguably been the most significant reason for the stagnation of the implementation of inclusive education in South Africa. Therefore, this article focuses on investigating the inclusive strategies used by teachers for the inclusion of learners, specifically those with disabilities such as autism spectrum disorder (ASD), in inclusive settings.

Autism spectrum disorder research is lacking in South Africa, and this study aims to contribute to this under-researched area. Indeed, accurate statistics on the prevalence of ASD in the country are still unavailable (Meiring et al. 2016). Autism spectrum disorder is the fastest-growing neurodevelopmental disorder worldwide (Akhter et al. 2018), and it is important that it is more fully understood, especially in the context of teaching and learning because with increased prevalence rates, teachers can expect to encounter more learners with ASD in their classrooms. The main addition that the study makes is to suggest ways in which some pedagogical strategies can be developed and used to ensure better outcomes for learners with ASD in South Africa. Much of the knowledge of these pedagogical strategies was obtained from teachers, speaking from positions of experiences where their strategies have been tried and tested.

## Literature review

### History of inclusive education in South Africa

The political history of South Africa has radically transformed over the last 25 years, from a situation that denied the needs and rights of the majority, in favour of a minority, to a democracy that enshrines equal rights to all. However, this equality is still not reflected in the educational landscape. This pertains especially to learners with disabilities who, during and after apartheid, have remained on the fringes of the education system (Naicker 2007; Ntombela 2011). Prior to 1994, aside from separation based on race, learners were also placed into separate schools because of mental and physical impairments and disabilities. This practice has continued in the ‘new’ South Africa despite the emergence of official legislation (Department of Education 1996, 2001) mandating the equalisation of educational opportunities for all children in the country. Education White Paper 6 (Department of Education 2001) in particular represents the best legislative effort to date through which the government expresses its vision for inclusive education (Du Plessis 2013). Thus, success in implementing this vision will translate into success in implementing inclusive education in South Africa.

Unfortunately, there has been little meaningful progress, as the latest report on the government’s progress on implementing EWP6 indicates that the majority of learners with disabilities still attend special schools (Chambers et al. 2017; Department of Education 2016). This demonstrates that the ‘paradigm shift’ in policies is not yet reflected in schools or in practice. According to Engelbrecht et al. (2016), the essential paradigm shift needed is one from the medical model to the social model of disability. The medical model views disability as an individual and deficit problem, necessitating placing learners with disabilities into separate, special education institutions as they are not considered to be educable alongside their peers (Naicker 2005). Conversely, the social model explores how society itself creates and reinforces negative perceptions of physical and mental disability and advocates for inclusive educational institutions, where all children are taught together. It is evident that the South African educational landscape is still largely managed according to special education principles, given the placement of most disabled learners into special schools (Department of Education 2016). However, arguably both models are insufficient in fully conceptualising disability and in developing practical strategies to assist impaired learners. The medical model’s major shortfall is its narrowness in ascribing difficulties associated with disability to the individual, whilst the general social environment remains largely unchanged and marginalisation persists (Dixon & Verenikina 2007). Although the social model primarily seeks to correct societal attitudes towards people with disabilities (Oliver 1996), it does not give enough attention to the scientific aspects of impairment, which, if understood, can lead to more informed ways of assisting people in line with the specific needs they have. There is also clearly a need to explore and develop an understanding of inclusive pedagogy, and the need to empower teachers to respond appropriately to a diverse range of learning needs, including those specific to particular impairments, if paradigm and practice shifts are to be supported (Walton & Bekker 2016).

The importance of teachers in the implementation of inclusive education cannot be ignored as ‘classroom educators will be our primary resource for achieving our goal of an inclusive education and training system’ (Department of Education 2001:18). South African teachers over decades have been mandated to follow policies and guidelines that were devised without their active involvement. For example, whilst the EWP6 development is said to have included the recommendations of teachers (Lomofsky & Lazarus 2001), subsequent policies were needed to give clearer classroom-based guidance. An attempt by government to meet this need was the introduction of the Guidelines for Inclusive Teaching and Learning (Department of Education 2010b) and the Guidelines for Full-Service Schools (Department of Education 2010a). These guidelines, however, remain generic and descriptive rather than providing practical strategies (Du Plessis 2013). Hence, we argue that it is necessary to explore the strategies and approaches used by teachers themselves, in diverse contexts, to support the inclusion of learners and specifically for this study, those with ASD, in order to understand how inclusive education may be better realised.

Thus, in this study, ASD, a neurodevelopmental syndrome that mainly affects people’s social interaction (Akhter et al. 2018), is the focus for exploring strategies and approaches used by teachers to support the specific learning needs of these learners.

### Autism spectrum disorder research in South Africa

Autism spectrum disorder rates have risen sharply in recent years, with North America, for example, having 1 in every 165 children diagnosed with autism (Lindsay et al. 2014). Dyer (2010) posits that the increase in diagnoses is partly because of improved technology and changes in the categorisation of children with ASD. According to Jick and Kaye (2003), more comprehensive studies suggest that hereditary and prenatal conditions of parents are by far the most common causes of ASD known to scientists. Similarly, Ametepee and Chitiyo (2009) argue that scientists generally point to genetics as the main source for ASD. However, most of this information has Western origins, and the prevalence in African countries is largely unknown, as there is very little ASD research originating from Africa, and the rest of the developing world (Ametepee & Chitiyo 2009). Springer et al. (2013) show that 94% of all articles published on ASD have European and North American origins. Chambers et al. (2017) argue that South African society in general lacks knowledge of ASD, and that there is little specific locally generated knowledge on supporting learning for learners with ASD because of the lack of research in the country, which strongly supports the need for studies like the present one to discuss ASD in a local-centred context.

Majoko (2017) argues that the description of characteristics associated with ASD is challenging, given the many individual differences amongst learners with ASD. We support this argument and acknowledge that not all learners with ASD can be described as experiencing identical challenges. However, some common challenges that may be experienced by learners with ASD have been described in the literature. Such challenges include difficulties in interacting with others, verbal and non-verbal communication challenges, a prevalence of repetitive behaviour and difficulties processing sensory input (Majoko 2017; Park, Chitiyo & Choi 2010). Difficulties with sensory input and discomfort with changes in routine make navigating a classroom setting difficult for many learners with ASD.

For learners with ASD fortunate enough to attend school, the challenge remains that many teachers struggle to support these learners to achieve reasonable educational outcomes, because of the lack of strategies and resources available for teaching learners with ASD (Van der Linder, Erasmus & Kritzinger). Teachers, especially in mainstream schools, face additional challenges as they are tasked with assisting learners with ASD alongside typically developing children, who themselves still have diverse needs. In addition, despite EWP6 (Department of Education 2001) prioritising changing the attitudes and competencies of teachers towards teaching learners with disabilities, some negative attitudes towards teaching learners with disabilities persist (Erasmus, Kritzinger & Van der Linde 2019; Nel et al. 2011; Swart et al. 2002). Research indicates, however, that whilst some negative attitudes still persist, many teachers in South Africa support in principle the idea of catering for learners with disabilities in mainstream schools and have favourable attitudes towards inclusive education (Donohue & Bornman 2015; Nel et al. 2011; Swart et al. 2002). What concerns teachers however is that they lack the skills to teach increasingly diverse learner populations, most notably those including learners with disorders such as ASD who present behavioural challenges (Erasmus et al. 2019; Tissot & Evans 2003).

Given the lack of resources in developing countries (Engelbrecht et al., 2016), cost-effective pedagogical approaches can contribute positively to assisting learners with autism. One technique is the use of visual aids such as placards. This is a relevant option given that most learners with autism learn best when observable materials are included (Tissot & Evans, 2003). Apart from being used as learning materials, visual symbols can also be placed around the school indicating time slots for specific activities, so as to avoid informing learners about tasks suddenly, which can potentially cause anxiety in ASD learners (Hansen et al., 2014). Furthermore, in order to enable teachers to effectively teach in diverse classes, Ntombela (2011) suggests that teachers should be given sufficient training before they start their careers and still more in-service training to remain cognisant of emerging challenges. As already highlighted, ASD is increasing in prevalence and is commonly assumed to require special education strategies and settings, and hence teachers need to know more about it and be equipped with strategies to teach learners effectively. This study aims to investigate how teachers adapt their teaching strategies for ASD in inclusive settings.

## Theoretical framework

### Inclusive pedagogical approach in action

In this article, the Inclusive Pedagogical Approach in Action (IPAA) framework (Spratt & Florian, 2015) is used as a theoretical framework. This framework originated from research into teachers’ inclusive responses to learners’ individual differences to enhance participation and avoid exclusion, marginalisation or stigmatisation (Florian & Black-Hawkins 2011). Three key principles that are considered essential to enacting inclusive pedagogy in the classroom underpin IPAA.

The first of these principles is that difference must be accounted for as an essential aspect of human development in any conceptualisation of learning. Inclusive pedagogy does not deny difference in learner learning but advocates responding to differences in a way that does not marginalise learners through teachers’ responses to meeting learner needs, by extending that which is ordinarily available to all (Florian 2010b). Teachers therefore need to reject deterministic views of ability, and the idea that the presence of some children will impede the progress of other children (Florian 2015). Deterministic views are associated with the belief that ability is predetermined and fixed and therefore unchangeable. This deterministic view of ability should be replaced by ‘transformability’, which is associated with a belief that all children can progress if provided with appropriate support and conditions. Transformability suggests that ability is not static but can be influenced through the actions of teaching and learning (Hart & Drummond 2014). Learners with ASD are different in how they comprehend information and communicate, and the IPAA perspective acknowledges that the difference exists. Nevertheless, the approach rejects the common hesitancy to teach them in inclusive settings because of assumptions of their lack of ability to learn the same content as non-ASD children. Inclusive Pedagogical Approach in Action, rather, advocates for pragmatic steps, beginning with teacher beliefs, to ensure that the school environment transforms for all learners to participate meaningfully at school.

The second IPAA principle is that teachers must believe that they can teach all learners, including learners with special educational needs, in their classroom. Teacher efficacy for inclusive education has been shown to be a key factor in the successful implementation of inclusive education and a key factor in determining teacher attitudes towards learners with special educational needs (Forlin, Sharma & Loreman 2014). A lack of teacher efficacy can have a negative impact on teacher acceptance of, and interaction with, learners with special educational needs. Essential to developing teachers’ belief that they are capable of teaching all learners is the view that learning difficulties are not problems located within the learner but rather challenges for the teacher to respond to, thus seeing learning difficulties as a professional challenge. This means that the teacher is committed to the learning of all and views the learning of all as a professional responsibility for which they are accountable. In this way, teachers commit to support the learning of all (Florian 2014). This contrasts with the traditional view that assumes that learning difficulties are located within the learners, and that they therefore require something else, from someone else, preferably somewhere else, to address their learning difficulties. Moreover, studies have shown that South African teachers understand the importance of teaching all learners in an inclusive environment, but they are not particularly confident in their abilities to teach learners with disabilities (Ravet 2018). This shows that a constraint to inclusive practice, when it comes to teachers, is their perception of their abilities, which needs to be supported by enhancing the development of pedagogical strategies that they feel confident drawing upon.

The third IPAA principle stresses the importance of continual professional learning and developing new strategies for working with others. This acknowledges that teachers need support in developing their understanding of inclusive pedagogy and how to enact this in the classroom (Florian 2010a). One such means of support is teacher collaboration, which is widely accepted as a key to inclusive practice (Ainscow 2014). Collaboration extends to the collaboration of teachers with parents, guardians and support professionals, as well as to encouraging collaboration amongst and between learners themselves and learners and teachers. This framework is thus relevant to the study’s objective of finding out how teachers, who have succeeded in including learners with ASD in their classrooms, were able to do so and to suggest ways of facilitating the exchange of skills with their less-experienced colleagues.

There has been some criticism of IPAA primarily regarding the complexity of considering levels of difference that may occur between learners. These levels of difference may present challenges to address individual learner differences within the whole class setting. Lindsay et al. (2014) conducted a study of teachers’ practices for including learners with ASD in mainstream classrooms and found that whilst teachers embraced inclusive pedagogy, they needed to use specific strategies for learners with ASD to manage behaviours. This use of specific strategies, focused on specific learners, could be considered exclusionary. It has been argued that continued development of the IPAA is required in order to address concerns related to avoiding targeted approaches for specific learners whilst still being mindful of incorporating goals of individualised learning plans where these are required. Despite this, we argue, however, that the IPAA provides a useful lens through which to consider teachers’ responses to learners with ASD, which is the focus of this article.

## Research questions

The research questions posed for this study are as follows:

- What are teachers’ experiences of teaching learners with autism spectrum disorder inclusively in the classroom?
- What strategies have led to teachers’ success in teaching learners with autism spectrum disorder in the three schools included in the study?

## Aims of the study

The main objective of the study was to understand the experiences of teachers teaching learners with ASD in the inclusive classroom. The study’s additional aim was to understand the strategies that teachers use to effectively include learners with ASD in inclusive educational settings. These strategies are then collated in the article to address Engelbrecht et al.’s (2016) concern that EWP6 (Department of Education 2001) is particularly lacking in specifying practical inclusive strategies, making it a policy with the right intent, but one that lacks direction on the practice of inclusive education in the classroom space.

### Methodology

#### Research approach

A qualitative interpretivist research methodology was used to give respondents the opportunity to explain their experiences and to give their views on the best educational provision for learners with ASD.

**Sampling:** The aims of the study required narrow purposive sampling, namely, teachers who have experience in teaching learners with ASD. Purposive sampling is found to be appropriate for qualitative research because a qualitative study seeks depth of experience and asks carefully selected participants to share their views and motivations. Merriam and Tisdell (2015:96) add that qualitative researchers seek ‘to discover, understand, and gain insight and therefore must select a sample from which the most can be learned’. A purposive sample was therefore appropriate for collecting data from select sources that are most likely to possess the relevant knowledge (Scott & Morrison 2006).

Three schools were selected as research sites for this study. These include one mainstream school, one full-service school and one special school. The following description of the individual schools gives more context to the study and reflects the considerations made in choosing each. Firstly, a mainstream secondary school located in a suburb, predominantly housing learners from middle to upper-income households, was selected. Although the school uses a mainstream curriculum, it pays a great deal of attention to special needs learners, as reflected in the description on its website. Secondly, a full-service school located in a township was chosen because it was transformed from a mainstream to a full-service school for the purpose of implementing inclusive education and is a good example of how far the advancement of inclusion has come. The neighbourhood surrounding this full-service school faces many socio-economic challenges, which compounds the difficulty that comes with teaching learners with impairments. Finally, to properly capture the role of special schools in the inclusive education objectives, a special school for learners with ASD was also included in the study.

The sample size across the three selected schools comprised seven teachers. Two teachers from the mainstream school, three teachers from the full-service school and two teachers from the special school were interviewed. Initially the intention was to interview at least 10 teachers. However, the 2020 coronavirus disease 2019 (COVID-19) pandemic, and strict lockdown measures, made it difficult to secure permission from principals to conduct research in their schools, despite virtual interviews being a possibility that was offered. The selection of teachers for interviews was contingent on them having taught learners with ASD in the past, or that they were currently doing so. The actual selection was done by the principals in each school who were requested to specifically identify teachers who have regular interaction with learners with ASD to be invited to participate in this study.

#### Data collection

Although the initial sample size was not secured, the objectives of this study were not significantly affected as it is a qualitative, interpretivist study concerned with understanding and exploring teachers’ experiences of, and strategies for, teaching learners with ASD. Here, Meiring et al. (2016:2) assert that ‘the sampling strategy for qualitative research is less concerned with the size of the sample, but more with the relevance of the sample’. Relevance, in this case, is determined by whether the interview structure allows for in-depth statements from the respondents, which provides comprehensive feedback that sheds light on their teaching experiences, along with their recommendations for policy development. The kind of interview format used was phenomenological interviews, which allow participants to detail their experiences regarding particular issues (Moustakas 1994). This was an appropriate method because the aims of the study were to understand the viewpoints of the participants.

Open-ended questions were used in the interview to ensure that respondents would not be limited in giving their opinions and experiences. Themes, based on the gap in South African research on ASD, and on areas in which inclusive education has seen slow progress, were developed into interview questions. These included the lack of knowledge about ASD, and concerns of teachers not having proper training to function in an inclusive education environment. Teachers were given an opportunity to clarify whether they feel confident in teaching learners with ASD, alongside typically developing children. Ten questions were asked, and each interview lasted for 30 min to allow for a comprehensive and detailed response from each participant. The following is one of the combination questions included in the interview schedule for teachers in their special school:

‘Are you confident in your abilities to attend to the needs of learners with autism? How do you think your confidence and ability levels would be affected if you taught them (ASD learners) in a mainstream school?’

This question allows the respondent to discuss their teaching efficacy, the experience they have gained in the special school and whether they think this has an impact on their effectiveness in a mainstream setting. The question allows for a range of possible responses, supporting Siedman’s (1998) assertion that open-ended questions enable the researcher to address key themes and also discuss other vital points, as they arise in the interview. An advantage of this approach is that commonalities in the responses of different teachers on themes that were not predetermined were noted, and the issues raised were then considered for further research. In essence, each question in the interviews would either shed light on any of the pre-set themes or allow the teacher to explore other issues and challenges that may not have been included amongst the initial themes.

Furthermore, open-ended questions meant that some of the themes emerged because of responses from the teachers. This supports Siedman’s (1998) claim that open-ended questions can address key themes and also develop additional themes and issues as they arise in the interview. An additional advantage to using open-ended questions is that research subjects are able to bring in new points of consideration that were not part of the initial focus of the study, but that have significance to the participants. Nind (2014) asserts that inclusive research should aim to give those who participate in the research enough control for them to determine what will be most meaningful and relevant to discuss.

### Ethical considerations

Ethical clearance to conduct the study was obtained from the University of the Witwatersrand Research Office Human Research Ethics Committee (Non-Medical) and the Gauteng Department of Education as well as the principals of the schools and the interviewed teachers. Furthermore, given the restrictions imposed by the South African government because of the pandemic, all data gathering activities strictly observed COVID-19 protocols. Participants were also given the option to participate virtually through online platforms if they did not want to be physically present for an interview.

## Data analysis

The interview data were processed through thematic analysis, which is defined as ‘the process of analysing data according to commonalities, relationships and differences across a data set’ (Gibson & Brown 2009:127). This process is also known as ‘coding’, which, according to Gibson and Brown (2009), involves forming categories that are used to describe general features of data in which different respondents raise similar views. Under this method, there are two different domains of codes, which are *a priori* codes and emergent codes. *A priori* codes are developed before data collection takes place and anticipate themes the research seeks to explore (Scott & Morrison 2006). These themes guide the main questions in the interviews, as they are structured to respond directly to the research questions of the study, ensuring that the main objectives are addressed. Emergent codes are constructs from the research participants; these emerge from the respondents during the interview, as they respond to the open-ended questions. Importantly, they add the subjects’ own voices to the research findings. To show the broadness of the coding process, Gibson and Brown (2009:133) write that emergent codes ‘emerge through the exploration of data … as distinct interests that were unforeseen in the original formulation of interests’. Numerous points emerged during the data collection process of this study. However, only emergent themes that held the most weight in terms of responding to the research questions are discussed in this article.

## Assumptions

As reflected in the *a priori* themes, the following assumptions were made prior to the collection of data. Firstly, that all the teachers involved in the study had at least a basic knowledge of EWP6, as it is the national blueprint on teaching learners with disabilities who, according to their schools’ credentials, are part of their learner population. Secondly, as the principals had affirmed, in the process of giving permission to conduct research in their schools, that they had learners with ASD, it was reasonable to assume that all the teachers interviewed had some experience of teaching learners with ASD. To ascertain this, the interview questions for the mainstream schools contained one question asking teachers to state whether they had any learners with ASD in any of the classes they taught. In the case of the special school, no such enquiry was necessary, as it is a specialised institution specifically for children with ASD.

## Presentation and discussion of findings

As stated in the methodology section, data analysis was conducted using thematic analysis, and the results are presented here under specified themes, namely (1) teachers’ understanding of ASD and strategies, (2) teachers’ views on the inclusion of learners with ASD and curriculum choices and (3) continuous development of teachers. As the data collection involved people, abbreviated pseudonyms were used as follows: each abbreviation signifies the kind of school the teacher was from (mainstream, full-service or special school), and the number in the abbreviation indicates the teacher in that institution:

- Mainstream school teachers: MT1 and MT2
- Full-service school teachers: FST1, FST2 and FST3
- Special school teachers: SE1 and SE2

### Teachers’ understanding of autism spectrum disorder and strategies

Teachers understood ASD as being a developmental disorder and as a personality type, such as being an introvert.

‘A developmental disorder affecting ability to effectively communicate and socialise.’ (FST3)

‘It is a situation or character of an individual human being who is an introvert, who fails to click well with others.’ (FST1)

‘If you will not talk to them, they will not talk to you … what happens when the child goes down the stairs, he is scared, locomotion, the whole system; it’s sensory disorder.’ (SE1)

‘A disorder with problems communicating, structural problems in the brain that doesn’t allow the child to understand figurative language, sarcasm and things like that, and quite often an inability to look someone in the eye.’ (MT1)

The explanations of ASD provided by participants above included descriptions of particular challenges faced, such as problems communicating, which aligns with similar descriptions in the literature of social interaction and communication challenges (Majoko 2017). Some complexities that characterise ASD were noted in teacher descriptions of ASD including sensory sensitivity and experiencing difficulty with understanding figurative language and sarcasm.

The teachers’ understanding of the complexities that characterise ASD is an important step in implementing appropriate teaching strategies that will support learning for these learners. One of the highlighted strategies was to boost leaners’ strengths, which reflects a particular phenomenon related to ASD. This is the observed phenomenon in children with ASD that they each have a certain trait, or knowledge area, where they surpass the levels shown by most of their agemates. The reason for this advantage, according to Timmons, Brietenbach and MacIsaac (2006), is caused by their compulsive interest in certain forms of knowledge, skills or objects. In recognition of this, MT1 gives a fitting teaching strategy:

‘Play to their strengths because you will find that quite often they have strengths that other learners do not have, and in a groupwork situation they become quite valuable.’

This strategy properly exemplifies the values of inclusion, as it enables the peers of learners with ASD to not only see but also benefit from the skills of those with ASD. An appropriate situation on which to embrace the strengths of ASD learners is groupwork:

‘Groupwork strategies also produce acceptance, excellency and understanding amongst learners at large.’ (FST3)

Acceptance is essential for inclusion, and the acknowledgement of each other’s strengths helps learners to function well collectively; therefore, groupwork as a strategy is vital and needs to be done with all the differences in the learner population in mind. Differentiated instruction is another strategy that teachers use to ensure that the learning of all pupils runs smoothly, and that all have equitable opportunities to learn. This pedagogy is described by Tomlinson (2005:263) as ‘a philosophy of teaching purporting that students learn best when their teachers effectively address variance in students’ readiness levels, interests, and learning profile preferences’:

‘… [*Y*]ou cannot dwell on one pedagogical strategy, it depends on the topic of a day, differentiation helps a lot.’ (FST1)

According to Algozzine and Anderson (2007:50), the priority ‘to the teacher who differentiates is providing a learning environment and opportunities that exclude no child’. Importantly, inclusive education is not merely a philosophy that seeks to cater for the needs of learners with impairments or those that are marginalised in any way, but one that serves all learners. Göransson and Nilholm (2014:207) define the term ‘inclusive education’ as the ‘creation of communities’ and differentiated instruction, through its principle of ensuring that the learning preferences of all learners are considered, which leads to the classroom becoming a community in which all members are equal in opportunities to learn. The participant quote above (FST1) also points to the flexibility of pedagogical approach enabled when using differentiation in the classroom and the multiple ways of being responsive that this opens up for pedagogical choices (Walton & Bekker 2016).

Another element to differentiated instruction noted in the interviews was to involve learners in the planning of the lesson and to allow them to choose their preferred method of learning. This is also important in activities that are not confined to the classroom as demonstrated in the following example:

‘I also teach LOE, which is like PE… the autistic kids are not necessarily team players, and if you were to force them to be there, it would be a challenge. They would tend to shy away and, you would be like that’s fine, do these exercises because you do need to get some exercise, but if you don’t want to play soccer, that’s fine.’ (MT2)

This strategy is effective in that allowing the learner with ASD a choice of activity does not bring about a different result from the one that the teacher intended, but through different means that are comfortable to each learner, the desired aim of getting exercise can be met. To involve learners in the planning and development of the lesson, scaffolding is seen to be a useful strategy as well:

‘The first thing is you need to scaffold the learning because of the inattention… they also want to know exactly what’s going to happen in this lesson… you need to explain the reason why this specific task and this specific lesson is important.’ (MT1)

Scaffolding, and other forms of making learners aware about what to expect in their lessons, is vital as it is done with the awareness that they can be anxious when abrupt changes are brought into their environments (Hansen et al. 2014). Along with these methods, there are pedagogical strategies that are based on the general challenge of learners with ASD to understand messages that others might regard as simple. Palko and Frawley (2009) assert that the majority of learners with ASD are visual learners, meaning that they understand communication better when it is presented to them in the form of visible depictions. Research has shown that most learners generally (with or without ASD) benefit from the use of visual aids (Tissot & Evans 2000). This means that the use of visual cues and teaching aids not only benefits learners with ASD but also presents advantages to typically developing children. The following extract from the interviews supports this view:

‘This is something that we try to do as staff consistently, and it’s to have a single hand gesture to indicate to the young person that he or she must have a breath now and let the others answer as well.’ (MT1)

Because of their compulsive obsession on phenomena that interests them, learners with autism may talk for a long time and not let others talk. Because of this behaviour, Ntombela (2011) suggests that many teachers find learners with ASD challenging as their behaviour may interfere with the learning of others. However, as seen in the example above using hand gestures as a visual cue, the behaviour can be regulated to maintain order in the lesson.

### Teachers’ views on the inclusion of learners with autism spectrum disorder

Many studies have shown that there is generally a positive stance amongst teachers towards inclusive education, with many supporting the placement of learners with disabilities into mainstream schools. However, these studies tend to be general, without any specific impairment being highlighted. This applies especially to those conducted in South Africa (see Dalton, McKenzie & Kahonde, 2012; Donohue & Bornman 2014; Engelbrecht, Nel & Tlale, 2014; Nel, Tlale, Engelbrecht & Nel, 2016; Ntombela 2011; Swart et al. 2002). Given this, it is important to understand from teachers themselves what placement they prefer for learners with specific challenges in the inclusive classroom to establish the teachers’ levels of readiness to assist such learners. Teachers were therefore asked if they specifically support the inclusion of learners with ASD into mainstream schools to establish whether this type of impairment has a bearing on teachers’ attitudes towards inclusion. Nel et al. (2011:77) argue that the attitude of a professional has an impact on their ability to produce desired results and adds that attitudes are ‘based upon previous experiences’.

Learners with ASD should not only be placed in mainstream schools because it aids them in societal integration. Rather, schools need to undergo the necessary reforms before they practise inclusion. The most crucial transformation efforts will be those focused on changing the attitudes of those who are likely to interact with children with ASD. This was found to be the case in the mainstream school, as revealed by the participants from that school. Mainstream school teacher 2 attested both to the value of inclusion of learners with ASD and the difficulty that comes with it:

‘I definitely think that there are advantages. However, there needs to be a sort of protected environment.’ (MT2)

This speaks about the adaptation of the social environment by changing the attitudes of other learners and teachers, thus removing prejudices about learners with impairments (UNESCO, 1994). Despite being in a mainstream school and having successfully taught learners with ASD in that environment, MT2 went on to express a personal preference that is not necessarily supportive of inclusion:

‘I think you’d have greater success in putting an autistic child in a special school.’ (MT2)

The teacher supported these remarks by pointing out the practical challenges of placement in most South African mainstream schools, which shows that the preference of special education is not a reflection on inclusive education being unrealistic, but that the South African context makes it so:

‘I think the reason for that is, I think when you go to really big mainstream schools, you’re looking at 1500 [*number of learners in a school*], you’re looking at 40–45 or more in a class. I think there is no teacher, amazing as they may be who’s going to help that child.’ (MT2)

The situation of overpopulation is indeed dire in many schools in South Africa, most notably in the most densely populated province of Gauteng. It is conceivable that many learners with ASD who are in school are not necessarily placed in special schools because their parents do not prefer mainstream education, but because the state of many mainstream schools in South African is not conducive for the education of their children. The issue of overcrowding was also pointed out by one of the full-service school (FST1) educators as a constraint on learning for children with ASD:

‘[*I*]t is very difficult to come up with the positive results because of overcrowding….’ (FST1)

The situation of large class size presents a challenge because it makes it difficult to assist learners individually, according to their needs. Despite the challenge of overcrowding, FST1 still insisted that the full-service school is the ideal environment for learners with ASD to learn in:

‘I think it is best to teach autistic learners together with mainstream learners because multi-learning will prevail for autistic learners. Groupwork strategies also produce acceptance, excellency and understanding amongst learners at large.’ (FST1)

Not only does this response show positivity towards inclusion, but it also shows concern for typically developing learners. Inclusive teaching is based primarily on this principle of conducting lessons in ways that benefit all learners, while at the same time avoiding their exclusion in the process of trying to include them (Messiou 2006, 2012). Of the three schools involved in the study, those from the private school showed a higher understanding and experience of this principle. Mainstream school teacher 1 relates an exemplary success story:

‘We had a young person that came here at an advanced age already, and we had to physically get him from the dark corners of the buildings every now and then… It took a year and a half for him to start talking, and then we couldn’t have him quiet, and then by the end of matric year he stood up and made a little speech in front of the whole school.’ (MT1)

The learner made significant improvements in communication, a social skill that is difficult for people with ASD to attain. An important aspect in the narrative above is that the teacher does not attribute the success to personal effort but to the work of the collective staff, as indicated by the repeated use of the word ‘we’. This is how inclusive education can be exemplified; it begins with the belief that any child can learn despite being impaired in some way. The Salamanca Statement and the efforts of inclusion that stemmed from it are anchored on this premise that ‘every child has unique characteristics, interests and abilities and learning needs’ (UNESCO 1994:6), and that they have a right to education that caters to their unique needs. This kind of education requires the collaborative action of all stakeholders, as demonstrated in the above example. When all teachers in a school work together, along with other non-teaching staff, children with ASD that would otherwise be seen as incapable of participating meaningfully can achieve satisfactory results (Causton-Theoharis et al. 2011).

With regard to whether learners with ASD should be taught a subject-specific curriculum or a skills-based curriculum, the answer is that for promoting inclusive education a flexible curriculum that covers both bases is needed. The need for a flexible curriculum to aid in the placement of learners with disabilities into mainstream schools was already proposed in 1994, as part of the United Nations Standard Rules for the Equalisation of Opportunities for Persons with Disabilities (United Nations Disabled Persons Unit 1994). If implemented across the country, this principle will set a different course for educators in mainstream schools; shifting them from the medical model mindset of finding a curriculum that learners with autism can fit into to changing existing curriculum practices to complement the different learners’ personalities and needs.

Amongst the issues raised by the participants is the choice of what kind of curriculum is appropriate for learners with ASD. The sector in which a child is educated has particular curriculum mandates that teachers follow, and this has a bearing on the results that can be attained in teaching learners with autism. In reality, one of the reasons that motivated the teachers’ choice of curriculum is the curriculum, which is taught in a school. As highlighted earlier, social awkwardness and slow development of communication skills are common amongst people with ASD. As a result, special schools, in particular the one that was involved in this study, work on a curriculum that is centred on the improvement of social skills. The school, as its website states, offers a curriculum that equips learners with ‘life skills required to help them function as independently as possible in activities of daily living’. Teachers interviewed at this school stated:

‘They can’t even do abcd, they don’t know because mainstream is mostly academic, and those kids would rather prepare for a skills background.’ (SE1)

‘There is a different mainstream curriculum; it’s a challenge to transfer them… I don’t recommend learners with autism to be in mainstream.’ (SE2)

The first statement does not acknowledge the diversity that characterises the autism spectrum; in the array of syndromes that make up the autism spectrum, there are different levels of severity (Tissot & Evans 2003). Some learners are high functioning, meaning that they have moderate to high intellectual comprehension despite having communication and language difficulties, so the generalisation of all ASD learners as incapable of academic success is inaccurate. What needs to be acknowledged and maximised is the potential of each child and a subsequent placement in a school that offers a curriculum suited to their abilities. This shows the attempt of the EWP6 objective to keep special schools in operation in an inclusive education system because on the other end of the spectrum, opposite to high-functioning learners are low-functioning ones who, indeed, are highly unlikely to benefit from a mainstream curriculum.

### Continuous development of teachers

The education system of South Africa, and by extension the world, is fluid, revealing different challenges at different times. This necessitates the training of teachers who are able to adjust their pedagogic strategies and respond to emerging issues in their field. Appropriate teacher training, therefore, does not end at the university or college stage but must be an ongoing process (McIntyre 2009). The importance of this was observed during the interview process, as the teachers highlighted that continuous professional development has boosted their teaching efforts, and that more needs to be done to ensure it. Experience was highlighted as a major contributor to teachers’ success in teaching learners with autism, as SE1 testifies:

‘It’s an ongoing thing, you learn all the time.’

University education sets an important foundation for teachers, but it is not enough to enable teachers cope with the reality of the teaching environment. Experience, as explained by the following responses, is vital for the development of teaching strategies:

‘I taught in private schools, and I taught in public schools, and I taught in public schools in very poor socio-economic environments… I think that has made me realise there is so many differences in each child in each class.’ (MT2)

‘I learned more here than I learned at university… I have worked here for 11 years if I don’t count this year. I did inclusive education in Honours but also, I was a high school teacher at grade 8 to matric, but then I went to a special school. I’ve got the experience; the experience helps a lot… It’s an ongoing thing, you learn all the time.’ (SE1)

‘Teaching is a labour of love and tolerance, as a teacher I must build a good relationship with all my learners. National policies contributed positively on inclusion to my abilities because workshops were organised to implement development to my teaching skills.’ (FST1)

These responses not only show the value of experience but also raise the question of how teachers without experience can thrive in an inclusive school with learners with ASD. This is where skills sharing becomes vital, and more experienced teachers can assist newly graduated colleagues in adapting to the classroom situation. Skills sharing is seldom highlighted in the EWP6 (Department of Education 2001) but was identified in this study as an effective tool for teaching learners with ASD. Special school teacher 2, for instance, suggested that teachers should train one another and share information about learners with ASD, and how to properly assist them:

‘The other teachers, I will also develop them because at the end they [*autistic learners*] won’t be in my class only… the whole school will understand.’ (SE2)

This method has also been successful in the mainstream school, as revealed by MT1, who explained how communication with one ASD learner is premeditated by all teaching staff in the school:

‘This is something that we try to do as staff consistently, and it’s to have a single hand gesture to indicate to the young person that he or she must have a breath now and let the others answer as well.’ (MT1)

This is a good strategy as it directly addresses one of the challenges associated with ASD. Learners with ASD are sensitive to slight changes in their surroundings, and this includes changes in how other people relate to them (Hansen et al. 2014). This collective strategy demonstrates what inclusive education can achieve. Here Causton-Theoharis et al. (2011) assert that children should not have to move to secluded learning spaces to learn but that regular learning changes should undergo the necessary changes to accommodate learners of all needs and backgrounds.

This is important because teachers’ success does not only depend on their own change of attitude but also on the extent to which they can change the attitudes of typically developing learners. Inclusive education is a whole school approach, and it is only by the collective effort of all members that it can be achieved.

## Conclusion

In investigating the experiences of teachers in teaching learners with ASD, and the strategies they implemented, it becomes clear that in terms of IPAA principles many examples of good practice were evident.

The first principle, that difference must be accounted for as an essential aspect of human development in any conceptualisation of learning, is clearly held by most teacher participants, as there is sensitivity in the descriptions of strategies utilised that indicate attention to including learners with ASD in ways that do not marginalise them. The second IPAA principle, which teachers must believe they are capable of teaching all learners, is held somewhat variably by the teacher participants of this study. Whilst there was a clear indication that these teachers viewed learning difficulties as a professional challenge and were committed to the learning of all, some still expressed views that learners with ASD would be better supported in special school settings. However, there was a clear commitment to the third IPAA principle of collaboration, with multiple examples of collaboration between teachers and in terms of developing strategies for collaboration amongst learners.

Teachers’ experiences of teaching learners with ASD have led to the use of strategies such as group work, differentiation, scaffolding and allowing for the choice of activity. Challenges to these strategies include large class sizes and a need for continued professional development. Teacher participants also felt that a flexible curriculum would support the ability to successfully enhance the participation of learners with ASD, alongside their peers.

In conclusion, we argue that the continued sharing of experiences and the ongoing development of collective pedagogic strategies that support the learning of all, including the learning of those with ASD, is of greater value than ongoing debates on which educational settings are most appropriate. The focus on placements and arguments for and against special school placements can be counterproductive. Supporting this Sansosti (2008) argues that focusing on where learners should be educated detracts from considerations on how best to support learning for diverse groups of learners. The end goal for inclusive education is an inclusive society, one in which opportunities are open to all people.
